# Venous Thromboembolism (VTE) Risk Assessment in an Older Adult Mental Health Inpatient Ward

**DOI:** 10.1192/bjo.2024.526

**Published:** 2024-08-01

**Authors:** Oluwatobi Ajewole, Margaret Ogbeide-Ihama

**Affiliations:** 1 Oxleas NHS Foundation Trust, London, United Kingdom; 2 King's College, London, United Kingdom

## Abstract

**Aims:**

This audit aimed to investigate how VTE risk assessments on one of our older adult inpatient units meet to the recommended standard by:

1. Assessing the compliance of admissions to the trust VTE policy, which is based on the corresponding National Institute for Health and Care Excellence (NICE) guideline.
2. Determining if VTE assessments were performed using appropriate clinical tools, as recommended in the policy, and correctly recorded in patient notes.

**Methods:**

All admissions to the ward (n = 77) within the one year from 01.06.2021 to 31.05.2022 were retrospectively reviewed for VTE assessments based on the abovementioned standards. Data was extracted from progress notes and ward round entries for completion of the VTE assessment during admission, documentation of the assessment tool, documentation of the VTE prophylaxis prescription if indicated, and reassessment of risks during admission.

**Results:**

This audit showed that only 3% of patients had a VTE assessment documented within the first 24 hours of admission. Overall, over a 10th of all patients never had an assessment, and of those who did, no one had the assessment tool used documented or uploaded on their clinical records. Also, of those who had a VTE assessment done, 5% were assessed to be at risk, and of these, only half had VTE prophylaxis prescribed.

**Conclusion:**

This audit showed the ward is essentially not meeting the standard for VTE risk assessment, with recommendations to incorporate VTE assessment as part of the clerking proforma and the medication charts, similar to the usual practice on physical health wards.

